# Footprints of Optimal Protein Assembly Strategies in the Operonic Structure of Prokaryotes

**DOI:** 10.3390/metabo5020252

**Published:** 2015-04-28

**Authors:** Jan Ewald, Martin Kötzing, Martin Bartl, Christoph Kaleta

**Affiliations:** 1 Research Group Theoretical Systems Biology, Friedrich-Schiller-Universität Jena, Leutragraben 1, 07743 Jena, Germany; E-Mails: jan.ewald@uni-jena.de (J.E.); martin.koetzing@uni-jena.de (M.K.); martin.bartl@uni-jena.de (M.B.); 2 Research Group Medical Systems Biology, Christian-Albrechts-Universität Kiel, Brunswiker Straße 10, 24105 Kiel, Germany

**Keywords:** protein assembly, dynamic optimization, optimal control, operon formation

## Abstract

In this work, we investigate optimality principles behind synthesis strategies for protein complexes using a dynamic optimization approach. We show that the cellular capacity of protein synthesis has a strong influence on optimal synthesis strategies reaching from a simultaneous to a sequential synthesis of the subunits of a protein complex. Sequential synthesis is preferred if protein synthesis is strongly limited, whereas a simultaneous synthesis is optimal in situations with a high protein synthesis capacity. We confirm the predictions of our optimization approach through the analysis of the operonic organization of protein complexes in several hundred prokaryotes. Thereby, we are able to show that cellular protein synthesis capacity is a driving force in the dissolution of operons comprising the subunits of a protein complex. Thus, we also provide a tested hypothesis explaining why the subunits of many prokaryotic protein complexes are distributed across several operons despite the presumably less precise co-regulation.

## 1. Introduction

Evolutionary derived optimality principles are an often used approach to understand and explain biological phenomena in metabolism [1,2], evolution of enzymes [3,4] or even in the arrangement of plant leaves [5]. Depending on the environmental conditions an organism faces, the proteomic machinery needs to be reorganized leading to the production of new proteins. Since there is a limited capacity for protein synthesis at any time in the life cycle of microorganisms [6], protein synthesis capacity is an important constraint in terms of the investment of time as well as available resources that shapes regulatory programs controlling protein synthesis [7,8].

One particular feature of the proteomic machinery of cells is the existence of protein complexes that are often assembled from more than one gene product. For example, according to the EcoCyc database [9], 1393 genes of *Escherichia coli* encode subunits of protein complexes and around 53% of them are associated with protein complexes with at least two protein entities (heteromers). The assembly of those complexes follows ordered steps and is not random due to the formation of energetically favorable subcomplexes [10]. This recent study provides evidence that the order of assembly is conserved through evolution by linking gene fusion events with assembly steps.

In this work, we investigate the optimal control of protein synthesis for the assembly into functional protein complexes. Since the stepwise assembly of protein complexes is known on an experimental basis only for a small number of complexes, like the 26s proteasome [11] or the RNA polymerase [12], we focus on general optimality principles behind protein complex synthesis and their influence on specific genomic features.

The genes encoding the subunits of protein complexes are often comprised in operons (in prokaryotes) [13] or in gene clusters (in eukaryotes) [14]. Amongst other explanations [15], there are two major reasons that are often cited to explain the advantage of arranging all proteins of a complex in a single operon [16]. These are the efficient co-regulation of genes [17] and the simplified horizontal gene transfer of functional protein complexes as a whole (selfish operons) [18]. While these explanations provide a reason for an evolutionary pressure to arrange all proteins of a complex in a single operon, the question, why a gene distribution of a protein complex across several operons is optimal, remains open. This question is of relevance since e.g., the majority (62%) all heteromers listed in EcoCyc are encoded by more than one operon.

In a previous work, we analyzed optimal regulatory strategies for the activation of metabolic pathways [8]. The observation was that two distinct strategies are optimal depending on the protein abundance and protein synthesis capacity. While a simultaneous activation of all enzyme is preferred if protein synthesis capacity is high or a small amount of protein needs to be produced. On the other hand, a sequential activation of enzymes is optimal if protein synthesis capacity is low or a large amount of protein needs to be produced. The latter activation strategy is also called just-in-time-activation and has been observed for several amino acid biosynthetic pathways in *Escherichia coli* [19,20]. The predicted optimal regulatory programs were validated through the analysis of the organization of genes of metabolic pathways into operons across a large number of annotated prokaryotic genomes. Optimizing the operonic organization in metabolic pathways is also an important component of Metabolic Engineering approaches. For example, expression vectors like ePathBrick, are designed to be adjustable in the operonic structure to provide an optimal regulation of metabolic pathways and maximum yield of their products [21]. Based on the linear flavonoid pathway of *Escherichia Coli* the system showed a much higher production when the single operon structure is disrupted by additional promoters and terminators [21].

Since the stepwise assembly of subunits of a protein complex into a functional unit shows strong similarities to the reaction sequences occurring in metabolic pathways, the aim of this work is to investigate whether principles that determine the optimal operonic organization of genes of a metabolic pathway are also relevant for the formation of protein complexes.

Intriguingly, for the flagellum of *Escherichia coli*, which comprises 20 different proteins, a sequential activation of proteins depending on the order in which the flagellum is assembled has already been observed [22]. To this end, we formulate a model of the synthesis and assembly of a protein complex containing four subunits and determine the optimal synthesis strategy of the constituting subunits using dynamic optimization. The optimal control of protein complex assembly through synthesis shows similarity to previous results. For limited protein synthesis capacity, we observe a sequential synthesis strategy and for a high protein synthesis capacity a simultaneous synthesis is optimal. To validate our predictions, we analyzed the operonic organization of a large number of protein complexes obtained from the EcoCyc [9] and the PDB databases [23]. In confirmation of the optimization results, we found a strong influence of protein abundance as well as protein synthesis capacity on the operonic organization of the genes constituting a protein complex.

## 2. Results and Discussion

### 2.1. Model Overview

**Figure 1:**
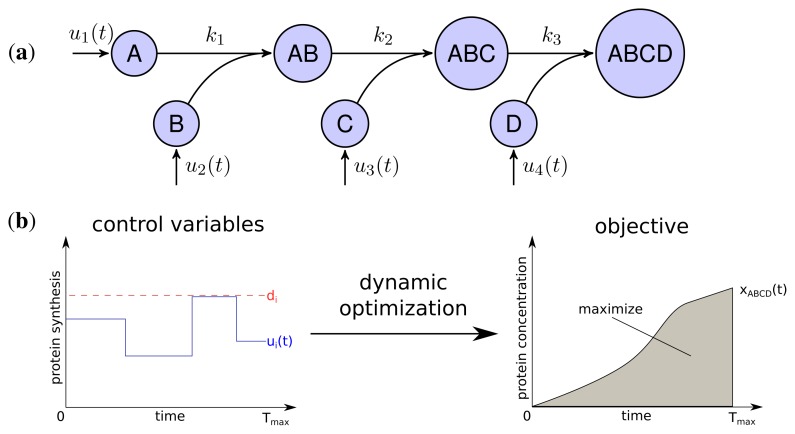
Scheme of the protein assembly model. (**a**) A,B,C and D are the subunits of the heterotetramer ABCD. The corresponding synthesis rates *u_i_*(*t*) are the control variables of the optimization problem and *k_l_* the kinetic parameters of the assembly steps; (**b**) Dynamic optimization determines synthesis rates *u_i_*(*t*) under the constraints of maximum individual synthesis rates *d_i_* to maximize production of the complex ABCD.

The developed model represents the linear stepwise assembly of a heterotetrameric protein complex with the subunits A, B, C and D (see Figure 1a). To model the protein complex assembly we use an ordinary differential equation (ODE) system with each assembly step *l* = 1; 2; 3 according a mass-action kinetic with the parameters *k_l_* (see also Methods). A biological example for an assembly network of this topology is the RNA polymerase of *Escherichia coli*, which is composed of 2 *α*, one *β*, one *β*′ and a *σ*-factor [12]. The assembly and the production of complex ABCD is controlled by the synthesis rate *u_i_*(*t*) of each subunit *i* which are determined by solving a dynamic optimization problem that maximize the amount of active protein complex over time (Figure 1b). Please note that through the utilization of the synthesis rates *u_i_*(*t*) we model protein production as a single combined process while a more explicit consideration would need to take into account transcription, translation and mRNA degradation. Since the abundance of a protein complex should be maximal for each time point to achieve its specific function efficiently, the objective is to maximize the integral of protein complex concentration over time:

(1) $$F(u)=\underset{u_{1}(t),\ldots ,u_{4}(t)}{\text{max}}\int_{0}^{T_{max}}x_{ABCD}(t)dt$$

with *x_ABCD_*(*t*) being the concentration of the final complex ABCD and *T_max_* = 15 as the considered time span of the simulation. Thus, we maximize the amount of active complex present in the course of the simulation. We assume an initial concentration of zero for all subunits and subcomplexes.

Two main constraints restrict the protein synthesis capacity of a cell. Firstly, each protein subunit *i* has a maximal individual synthesis capacity *d_i_*, which is influenced, for instance, by maximal copy numbers of mRNAs, translation efficiency and other rate limiting factors [24].

(2) $$0\leq u_{i}(t)\leq d_{i}$$

Secondly, we assume a maximal total protein synthesis capacity *d_total_* which is influenced, for example, by the number of free ribosomes and tRNA concentrations, as well as the total amount of competing mRNA of other genes in a cell:

(3) $$0\leq \sum_{i=1,\ldots ,4}u_{i}(t)\leq d_{total}$$

Both, total and individual synthesis capacities are varying for different organisms and environmental conditions. We observed, that only the relation between both constraints influences the optimal synthesis strategies (see Supplemental Figure S1) and therefore set *d_total_* = 1 and varied *d_i_*. The relation of the constraints *d_total_* and *d_i_* is studied in the next section and is followed by the analysis of the influence of kinetic parameters on the optimal synthesis strategies. As indicated, we also determined the impact of non-equal individual synthesis rates by randomization of *d_i_* for each subunit (see Methods) and tested their influence on the synthesis strategies.

*Adv*(*u_optimal_*), the advantage of the optimal production strategy over a simultaneous activation strategy was determined by comparing the objective function value for the optimized solution (*F*(*u_optimal_*)) against the objective function value assuming a simultaneous activation (*F*(*u_simultan_*)):

(4) $$Adv(u_{optimal})=1-\frac{F(u_{optimal})}{F(u_{simultan})}$$

### 2.2. Protein Synthesis Capacity Influences Optimal Synthesis Strategies

We investigated the influence of the relation between individual and total synthesis capacity by stepwise increasing the individual synthesis capacity *d_i_* from 0.25 to 1 (with *d_total_* = 1) with uniform kinetic parameters.

**Figure 2:**
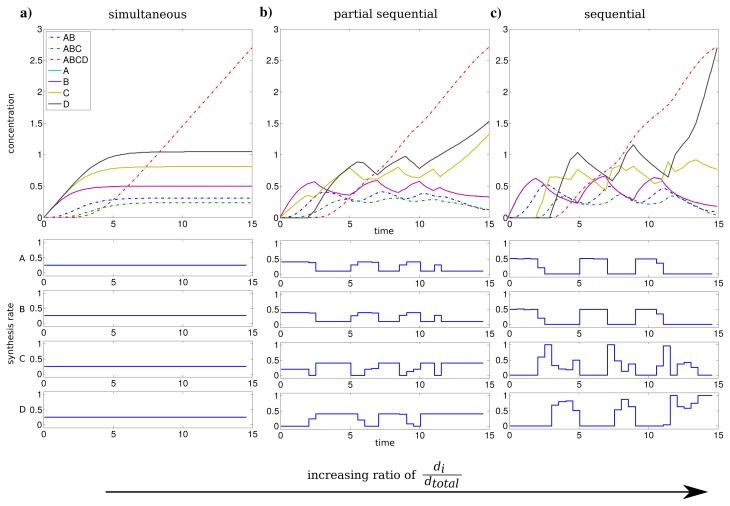
Time course of protein concentrations and synthesis rates for the individual synthesis rates *d_i_* 0.25 (**a**), 0.4 (**b**) and 1 (**c**). Subunit concentrations in solid lines and (sub)complexes in dashed lines.

For an individual synthesis capacity of *d_i_* = 0.25, the sum of *u_i_*(*t*) can not exceed the total synthesis capacity of *d_total_* = 1 and all subunits can be synthesized at the same time. The resulting optimal profiles are identical and subunits are synthesized simultaneously (see Figure 2a). However, when the individual synthesis capacity *d_i_* = 1 is equal to total synthesis capacity *d_total_* = 1, we observe a shift to a just-in-time similar strategy or here referred as sequential assembly strategy to be optimal (see Figure 2c). The stepwise assembly is reflected here in the order of protein synthesis with subunits A and B produced initially, followed by the synthesis of C and subsequently subunit D. The intermediate individual synthesis capacity *d_i_* = 0.4 leads to a partial sequential synthesis of subunits (see Figure 2b). This is expressed by the overlap of synthesis of proteins A, B and the subsequent simultaneous synthesis of C and D. The partial sequential synthesis is caused by the low individual synthesis limit of *d_i_* = 0.4, which implies that the synthesis of only two subunits are not sufficient to use the total synthesis capacity of *d_total_* = 1. Therefore the remaining capacity is used in the optimal control to produce the next subunit in the assembly order.

We compared the advantage of a sequential synthesis strategy to a simultaneous synthesis and found that the sequential strategy increased the amount of assembled protein complex by 2%–3% for *d_i_* = 0.4 and *d_i_* = 1 in contrast to a simultaneous synthesis.

Interestingly, the order of protein synthesis is principally repeated after the induction of the synthesis of the last protein (here D). These observations result in oscillating-like behavior with a repeated synthesis of consumed proteins in a conserved way which is basically similar to the corresponding initial protein synthesis. This can be interpreted as a production in packets in contrast to a constant stream of production as it is observed for low individual synthesis capacity. On the molecular level such a production behavior could be achieved through transcriptional bursting [25].

The considerations above are mainly based on the first time units, because they are representing the activation and a steady build up phase of the protein complex. For the last time units, we observe a strong synthesis of subunit C and D to maximize the amount of the final complex at the end of the time span. However, this effect is mainly an artifact of the requirement of a finite final time *t_f_* for the optimization procedure.

### 2.3. Kinetic Parameters of Assembly Steps Influence the Advantage of Sequential Synthesis

Since there might exist differences in the speed in which different subunits assemble into a complex, for instance due to the size of the interfaces between the subunits [10], we investigated the influence of the kinetic parameters of the assembly on protein complex production. To this end, we performed the optimization with hundred randomly chosen kinetic parameter sets with *k*_1_*, k*_2_*, k*_3_ ∈ [0*,* 2] for an individual synthesis capacity of *d_i_* = 0.75 and a total synthesis capacity of *d_total_* = 1. We compared the optimal solutions with non-uniform kinetic parameters to an assumed simultaneous synthesis with the same kinetic parameters to determine the advantage of the optimal solution.

**Figure 3:**
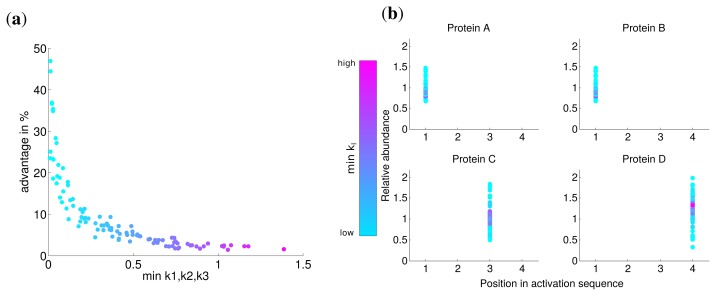
(**a**) Advantage of optimal solution compared to an assumed simultaneous synthesis of subunits in relation to the minimal kinetic parameter of each parameter set; (**b**) Position in activation sequence compared to the relative abundance for the same parameter sets as in (**a**). In both figures colors represent the minimal kinetic parameter.

The advantage for all optimization runs is visualized in Figure 3a and shows the benefit with respect to the lowest kinetic parameter of the set. This reveals a fast nonlinear increase of the advantage of the optimal solution (from 5% to 50%) in contrast to a simultaneous synthesis in particular for slow assembly steps indicated by small *k_i_* values. For larger minimal values of *k_i_*, the advantage is at the same order as for uniform kinetic parameters (about 3%). The correlation between the minimal kinetic parameter and the advantage is strong (Spearman correlation *r* = −0.965, *P* = 0) and suggests that especially slow assembly steps lead to the optimality of a sequential synthesis. Intriguingly, the activation order is the same for all random kinetic parameter sets (see Figure 3b). Our results show that the low speed of the assembly of specific subunits is compensated by a higher abundance of the corresponding subunits through a longer production (see also Figure S2).

### 2.4. Differences in Subunit Synthesis Capacity Determine the Order of Synthesis

The individual synthesis capacities are unlikely to be uniform for all subunits, because of differences in the speed of protein folding, differences in subunit sizes, translational efficiency of the corresponding mRNAs and other rate limiting factors [24]. To study the influence of these variations in our model, synthesis capacities *d_i_* were randomly chosen in [0,1] with a defined sum:

(5) $$\sum_{i=1}^{4}d_{i}=3$$

to be comparable to an uniform individual synthesis capacity of *d_i_* = 0.75.

For the comparison of the optimal solution to a simultaneous subunit production, the latter one has to be adjusted to fulfill the random individual synthesis capacities. This is done by limiting the synthesis rate to *u_simultan_* = *d_i_* if *d_i_* *<* 0.25, and then equally distributing the free capacity over the proteins with a higher individual synthesis capacity.

**Figure 4:**
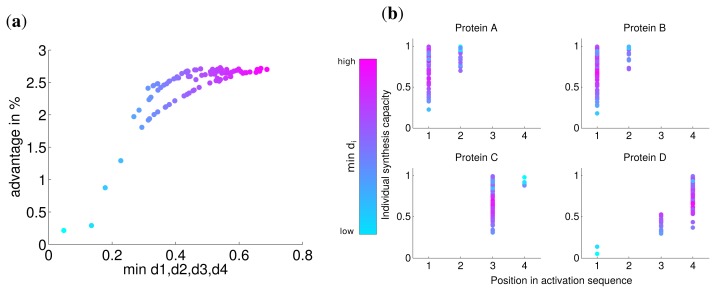
(**a**) Advantage of the optimal solution compared to a simultaneous synthesis of subunits; (**b**) Position in activation sequence compared to the individual synthesis capacity for the same parameter sets as in (**a**). In both figures colors represent the minimal individual synthesis capacity.

Analogous to the analysis in the last section, we calculated the advantage of the optimal solution and observed that the lowest value of *d_i_* has a strong correlation with this advantage (Spearman correlation *r* = 0.774 *P* = 0). For very low values of *d_i_* the advantage is nearly zero (Figure 4) and approaches the similar advantage of nearly 3% for high values of *d_i_*. This indicates, in accordance with our previous results, that for a very low synthesis capacity a simultaneous synthesis strategy is optimal.

The analysis of the activation order and synthesis rates of the individual subunits are depicted in Figure 4b. and shows that different individual synthesis capacities cause a reordering in the activation sequence. For instance, for some parameter sets the last subunit D is activated first and subsequently subunits A and B. The reason for this observation is that a subunit with a very low synthesis capacity is a bottleneck for the whole complex assembly and is produced at a maximal rate the whole simulated timespan (Supplemental Figure S3). At the same time, a high individual protein synthesis capacity can lead to the optimality of a later activation in the sequence as can be observed for subunit A and C for cases with high individual synthesis capacity. Hence, while a low individual synthesis capacity can lead to the optimality of a earlier activation, a high individual capacity can lead to the optimality of a later activation in contrast to the order of protein complex assembly.

### 2.5. Signatures of Optimal Synthesis Strategies are Reflected in the Operonic Organization of Protein Complexes

The analysis of the protein assembly model reveals a strong influence of protein synthesis capacity on the optimal assembly strategy. For a strongly limited protein synthesis, our dynamic optimization approach shows that a sequential synthesis is optimal. Whereas a simultaneous synthesis of subunits is optimal, when total synthesis capacity is high. These results are consistent with a previous study in which optimal activation strategies of metabolic pathways were studied [8]. To test whether these synthesis strategies are indeed used *in vivo*, we derived, in similarity to [8], several hypotheses on the operonic organization of protein complexes depending on individual and total protein synthesis capacity.

To this end, we assume that protein complexes and their subunits are simultaneously transcribed if they are present in a single operon. Using this assumption, we can test the validity of the optimization results by studying the influence of several factors influencing individual and total protein synthesis capacity on the size of operons. Similar to our previous study, these factors are the number of protein coding genes, the genomic copy number of ribosomal RNA (rRNA) genes and abundance of the protein complexes. While there are many other factors influencing protein biosynthetic capacity such as mRNA degradation rates and translation efficiency, this type of data is not readily available for a large number of organisms thereby precluding its utilization for the validation of our hypotheses.

For the validation of our optimization approach we consider three genomic features that have a strong influence on protein biosynthetic rates: the number of protein coding genes in a genome, the copy number of ribosomal RNA operons and the abundance of the protein complex. More information on how these features influence protein biosynthetic capacities can be found in [8]. Considering these features, we predict that a higher number of protein coding genes, which result in a potentially reduced synthesis capacity for the protein complex, lead to a smaller size of protein complex operons (hypothesis 1). Moreover, we expect that a higher copy number of rRNA genes, which increases protein synthesis capacity, results in a bigger size of protein complex operons (hypothesis 2). Furthermore, increased abundances of protein complexes should increase the corresponding individual synthesis capacity and, in consequence, the operons should be smaller in size (hypothesis 3).

To evaluate our hypotheses we studied the operonic structure of 550 prokaryotes listed in the MicroCyc database [26] and calculated the number of genes per operon (GpO) as an indicator for operon size (see Methods). The above postulated hypotheses were tested with the calculation of a partial Spearman correlations for each hypothesis. In all organisms we determined homologs of the protein complex, we correlated the GpO values with each hypothesis quantity while controlling for the other hypotheses (for details see Methods and Table 1). As basis for protein complexes we used two independent data sets. The first consists of all heteromers listed in the EcoCyc database with at least three distinct subunits. The second is derived from the PDB database and covers all heteromers with at least three distinct subunits. In the latter one, protein complexes are combined to avoid a bias due to an overrepresentation of particular protein complexes (mainly ribosomal structures, see Methods and Supplemental Figure S4).

**Figure 5:**
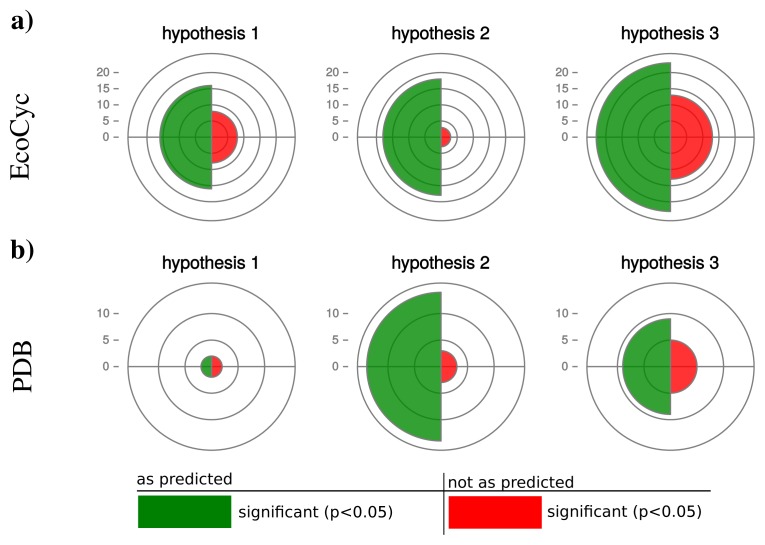
Summary of Validation results. The number of protein complexes confirming (green) or rejecting (red) our hypotheses are depicted as polar area diagrams where the size represents the count number. In (**a**) the results based on the protein complexes retrieved from the EcoCyc database and in (**b**) from the PDB database are displayed.

We retrieved 159 protein complexes with at least three entities from the EcoCyc database. For 100 protein complexes we found homologs in at least 50 organisms and could calculate a partial Spearman correlation. Table 2 displays the results for selected protein complexes and Figure 5a. illustrates a summary of confirmed and rejected hypotheses across all tested protein complexes (a detailed list is given in Supplemental Table S1 and S2). The table excerpt shows that our hypotheses are particularly confirmed by protein complexes which are shared by most prokaryotes, e.g., RNA-polymerase, ATP-synthase or chaperone systems. In general for hypothesis 1, we observe 16 protein complexes confirming our prediction and 8 protein complexes rejecting our hypothesis (significant correlation in the opposite direction). Also for hypothesis 2 and 3 the majority of protein complexes (hypothesis 2: 18, hypothesis 3: 23) confirm and the minority (hypothesis 2: 3, hypothesis 3: 13) reject our predictions.

The additional data set of protein complexes retrieved from the PDB database comprises 5250 heteromers with at least three protein entities. For 121 complexes we were able to find homologs in at least 50 organisms and calculated partial Spearman correlations between operon sizes as well as genomic features influencing protein synthesis capacity. Because the data set covers mostly ribosomal structures, we combined those protein complexes. Thereby we obtained 27 independent protein complexes.

For those, hypothesis 1 is confirmed by only 2 and rejected by 2 complexes (Figure 5). Hypotheses 2 and 3 are strongly confirmed by the majority of the investigated complexes (hypothesis 2: 14, hypothesis 3: 9) and rejected by fewer cases (hypothesis 2: 3, hypothesis 3: 6).

Interestingly, hypothesis 2 shows a better accordance with our prediction than hypothesis 1 and 3 with hypothesis 1 being significant for the smallest number of protein complexes. This observation can be explained by the many and diverse influences on the number of protein coding genes in an organism (hypothesis 1) or the static estimation of protein abundance by the CAI (hypothesis 3). The abundance of proteins is changing dynamically depending on environmental conditions and therefore influences the protein synthesis capacity of a cell. Moreover, environmental conditions have a strong influence on the protein biosynthetic capacity of a cell. Hence, the actual operonic organization can be considered as a compromise in operonic organization across the potential number of environments an organism faces.

Both data sets show that protein synthesis capacity influences the assembly strategy of protein synthesis and hence the genomic organization of genes. A sequential synthesis, caused by a low protein synthesis capacity, leads to smaller operon sizes. On the other hand, if protein synthesis capacity is high, the optimality of a simultaneous synthesis leads to larger operon sizes. Hence, also on the level of protein complexes protein biosynthetic capacities are an important factor in the formation and dissolution of operons. This observation is further emphasized by a recent study about the optimal genomic organization of the nitrogen fixation gene cluster of *Klebsiella oxytoca* [27]. Our results provide an explanation for the unexpected result that the disruption of operons leads to a higher production rate of active forms of nitrogenase in *Klebsiella oxytoca* and the transferred cluster in *Escherichia Coli*.

### 2.6. Acetyl-CoA-Carboxylase as an Example of Optimal Synthesis Strategies and their Implication for Operonic Organization

As an illustration of our predictions and validation results, we discuss in this section the genomic structure of the acetyl-CoA-carboxylase in *Escherichia coli* K12, *Staphylococcus areus* COL and *Peptoclostridium difficile* CD196 in more detail.

**Figure 6:**
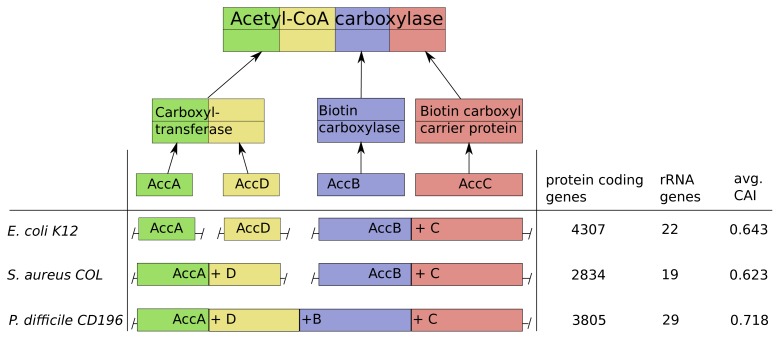
Acetyl-CoA carboxylase composition and operonic structure in three prokaryotic genomes. The colors and number of blocks represent schematically the subunit structure: [(*AccA*)_2_(*AccD*)_2_][(*AccC*)_2_][(*AccB*)_2_]. Next to the operon structure in each organism, the three genomic features influencing the protein synthesis capacity in the organism are showed.

Acetyl-CoA-carboxylase comprises three subcomplexes, which are assembled from four proteins AccA,-B,-C,-D (Figure 6). The subcomplex carboxyltransferase is composed of two subunits of the proteins AccA and AccD. The other subcomplexes biotin carboxylase and the carrier protein correspond to the proteins AccB and AccC [28]. Additionally, acetyl-CoA-carboxylase is of high interest due to its function as key player in the biosynthesis of fatty acids and is present not only in prokaryotes, but also in slightly different forms in plants, fungi and animals [29]. The multi-subunit complex catalyzes the first step of fatty acid synthesis by carboxylation of acetyl-CoA to malonyl-CoA and is strictly controlled via autoregulation of the AccBC operon [30–32]. To introduce lipid overproduction in biotechnological production organisms, the control and synthesis of acetyl-CoA-carboxylase is a main target for bioengineering and our results can provide avenues to improve its production by changing the operonic structure to achieve the accordingly optimal assembly strategy during induction of overproduction [33].

The operonic structure of acetyl-CoA-carboxylase coding genes varies across the 550 investigated prokaryotes. The three different forms we observed, are exemplarily depicted for *E. coli* K12, *S. areus* COL and *P. difficile* CD196 in Figure 6. In *E. coli* K12 the four coding genes are located in three operons and the genes AccB and AccC are located in the same operon, which is common in all other species. This is in line with our optimization results, due to the low number of rRNA genes (22) and high number of protein coding genes (4307), which results in a comparably low protein synthesis capacity and a sequential synthesis as the preferred strategy (Figure 6).

The pathogen *P. difficile* CD196 on the other hand has a lower number of protein coding genes (3805) and higher number of rRNA genes (29). This implied higher protein synthesis capacity corresponds to the fusion of all genes into a single operon, because a simultaneous synthesis should be preferred.

The methicillin, tetracycline and and streptomycin resistant *S. areus* COL strain has two operons coding for the acetyl-CoA-carboxylase subunits (AccAD and AccBC). This structure is explainable by the intermediate protein synthesis capacity, due to its low number of protein coding genes (2834) and rRNA genes (19). The formation into two operons fits with our optimization results, where we observed a partial sequential synthesis with a combined synthesis of the third and fourth subunit. The discussed relations between synthesis capacity and operon structure of the acetyl-CoA-carboxylase are mainly based on the number of protein coding genes and rRNA genes. This is due to the fact, that the CAI should have only a minor influence here, since it does not vary much between *E. coli* K12, *S. areus* COL and *P. difficile* CD196.

## 3. Conclusions

In our work, we integrated results from dynamic optimization of a protein assembly model with large-scale genomic data across several hundred prokaryotes. We could show how assembly strategies of protein complexes are linked to the formation of operons. The study of a protein assembly model identified a transition in the strategy of subunit synthesis from sequential to simultaneous depending on the protein synthesis capacity of a cell. We validated these results by determining footprints of the assembly strategy in the operonic structure of several hundred prokaryotes. For a large number of protein complexes, we could show that an arrangement in a single operon is favored if protein synthesis capacity is high and simultaneous synthesis of subunits is optimal. *Vice versa*, a scenario with a sequential synthesis strategy, which is optimal for low protein synthesis capacities, induces the separation of the proteins of a complex into individual transcription units.

These results are comparable with our previous results for metabolic pathways, which show the same strategies and their impact on the operonic structure of prokaryotes. Both results extend our understanding of evolutionary forces driving operon formation and dissolution. Most hypotheses explain why operons have an advantage like better co-regulation or easier horizontal gene transfer (selfish operons). In contrast, through our work we were able to identify factors that lead to the optimality of arranging co-regulated proteins in separate operons. Moreover, our findings also provide guidelines for metabolic engineering to optimally design operons containing protein complexes of interest when transferred from one species into a production strain.

## 4. Methods

### 4.1. Protein Assembly Model

In the model, all four subunits (A, B, C, D) have the initial concentration *x*(0) = 0 and are produced with a individual rate of *u_i_*(*t*) while forming the two intermediate complexes AB, ABC as well as the final complex ABCD. The assembly is modeled as a chemical differential equation system and each step is assumed to follow a mass-action kinetic with the kinetic parameter *k_i_*. The concentrations (arbitrary units) are captured in the vector *x*(*t*) and the differential states *ẋ* (*t*) are defined by the corresponding difference of synthesis and consumption:

(6) $$x(t)=\left(\begin{array}{c} x_{1}(t)\\ x_{2}(t)\\ x_{3}(t)\\ x_{4}(t)\\ x_{5}(t)\\ x_{6}(t)\\ x_{7}(t) \end{array}\right),\;\;\;x(0)=\vec{0}\;\;\;\dot{x}(t)=\left(\begin{array}{c} u_{1}(t)-v_{1}(t)\\ u_{2}(t)-v_{1}(t)\\ v_{1}(t)-v_{2}(t)\\ u_{3}(t)-v_{2}(t)\\ v_{2}(t)-v_{3}(t)\\ u_{4}(t)-v_{3}(t)\\ v_{3}(t) \end{array}\right)\;\;\;\cdots \;\;\;\begin{array}{c} \text{A}\\ \text{B}\\ \text{AB}\\ \text{C}\\ \text{ABC}\\ \text{D}\\ \text{ABCD} \end{array}$$

The velocities *v_l_*(*t*) are calculated according to a mass-action kinetic with *l* as the index of the assembly step:

(7) $$v_{l}(t)=k_{l}\;x_{2l-1}(t)\;x_{2l}(t),\;l=1,\ldots ,3$$

As in our previous work on metabolic pathways [8], the overall synthesis rate of protein is constrained. Therefore a total maximum synthesis capacity *d_total_* is introduced:

(8) $$0\leq \sum_{i=1,\ldots ,4}u_{i}(t)\leq d_{total}$$

Moreover, we assume an individual synthesis constraint of each component *i*, *d_i_* [8]:

(9) $$0\leq u{\left(t\right)}_{i}\leq d_{i}$$

Under these constraints the objective is to maximize the production of the final protein complex ABCD *x*_7_(*t*) over a fixed time span *T_max_* by finding the optimal program for protein synthesis and assembly:

(10) $$F(u)=\underset{u_{1}(t),\ldots ,u_{4}(t)}{\text{max}}\int_{0}^{T_{max}}x_{7}(t)dt$$

The model contains several parameters (see Table 3), which were tested for their influence. The decision variables *u_i_* are handled by the solver and are limited by a lower bound of zero and by individual synthesis capacities *d_i_*. The total synthesis capacity is fixed to *d_total_* = 1 and the individual capacities *d_i_* were tested in a range of [0.25*,* 1]. The kinetic parameters are either fixed *k_l_* = 1 or for the parameter test randomly chosen in the range *k_l_* ∈ [0*,* 2]. The simulated time span does not change the results and is simply fixed for all optimization runs to *T_max_* = 15.

### 4.2. Dynamic Optimization and Analysis of Simulation Results

The formulated nonlinear dynamic optimization problem is characterized by continuous, time dependent control and state variables. To solve the optimization problem, a quasi-sequential approach is used with an extension of Bartl *et al*. [34] to handle approximation errors and moving finite elements. Since this method is gradient based, we solved the problem one hundred times for each parameter set with random initialization of the decision variables *u_i_*(*t*). For subsequent analyses, the run with the best objective function value is used.

The solutions of optimizations are analyzed in a first step by visualizing the time course of protein concentrations and synthesis rates. Additionally, the advantage of the optimal solution to a simultaneous synthesis strategy is calculated and possible correlations with the randomized parameters are investigated. The simultaneous strategy was chosen as a reference and is defined by a constant synthesis rate *u_simultan_*(*t*) = 0.25. If the individual synthesis capacity *d_i_* of a subunit is lower than 0.25, the synthesis rate *u_simultan_* of this subunit is limited to *d_i_* and the remaining free synthesis capacity is equally distributed over the other subunits. The advantage of an optimal synthesis strategy *u_optimal_* is calculated as:

(11) $$Adv(u_{optimal})=1-\frac{F(u_{optimal})}{F(u_{simultan})}$$

To quantify the activation sequence of subunits, the activation time is calculated as the time *t_a_* when the synthesis of a subunit in sum exceeds its capacity:

(12) $$t_{activation}(i)=\underset{t_{a}}{\text{min}}:\int_{0}^{t_{a}}u_{i}(t)\;dt>d_{i}$$

Based on this, the subunits are ordered by the activation time *t_activation_*(*i*) and their rank is defined by the position in the activation sequence. The rank is plotted against the relative abundance or the individual synthesis capacity to reveal a possible correlation. The relative abundance of a protein is calculated as the maximum concentration in the first ten time units normalized to the average of 1 for each parameter set. The first ten time units are used, because the concentrations of the subunits in the last time units are not representative due to the limited final time.

### 4.3. Validation Process

To validate our hypotheses about the influence of protein synthesis on the genomic structure, we used data of protein complexes in many organisms with known operon structure and protein synthesis capacity. For protein complexes we used two independent data sets. The first was retrieved from the EcoCyc database [9] covering all protein complexes with at least three protein entities present in *E. coli*. The same was done for the gathered protein complexes from the protein structure database PDB [23], which is the second data set.

The orthologs of the subunits were determined using the Orthologous MAtrix (OMA) database [35] and were mapped to the proteins listed in Microscope database [26]. For the proteins of the Microscope database, we could use existing data from previous studies about the operonic structure and the codon adaption index (CAI) of the corresponding genes in 550 prokaryotes. Also we reused the number of protein coding genes and the number of ribosomal genes as estimate of protein biosynthesic capacity.

We used the operonic structure to calculate the number of genes per operon (GpO) for each complex in each organism, which is defined as the number of subunits divided by the number of operons the protein complex is synthesized from. The analysis is performed for protein complexes with orthologs in at least 50 species, where 50% of the subunits are found as orthologous. For calculating the Spearman correlation between the GpO and the hypotheses we corrected for organism specific features by determining the partial correlation of the GpO values and the investigated hypothesis while controlling for the other hypotheses and the average GpO of each organism, as depicted in Table 1. The resuling *p*-values are corrected for multiple testing at a false-discovery-rate of 5% by the Benjamini-Yekutieli procedure [36].

During the analysis, we observed that the protein complexes retrieved from the PDB database correspond mostly to ribosomal structures. To avoid bias we grouped protein complexes via hierarchical clustering according to their subunit composition. As a simple metric between two protein complexes with subunit sets *A* and *B* we define:

(13) $$d(A,B)=\frac{\left|A\backslash B\right|}{\left|A\right|}+\frac{\left|B\backslash A\right|}{\left|B\right|}$$

as the distance between two complexes. We used the resulting dendrogram (Supplemental Figure S4) to merge overlapping protein complexes and reduce bias in the PDB dataset. The correlation of a cluster is calculated as the average of combined protein complexes and *p*-values combined by applying the Fisher method.

## Figures and Tables

**Table 1:**
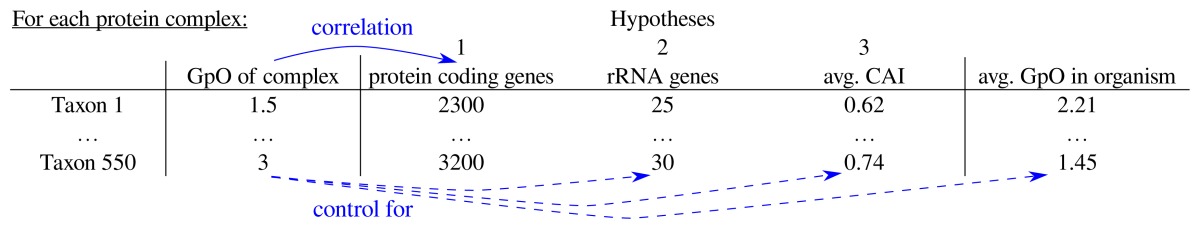
Determination of partial correlations (values chosen arbitrarily). Arrows in blue show the procedure of the determination of partial correlations for hypothesis 1. Spearman correlation between GpO and Hypothesis 1 is calculated while controlling for the other quantities (dotted arrows).

**Table 2:** Excerpt of validation results. The table shows the partial correlation for the three postulated hypotheses between operon size and factors influencing protein synthesis capacity (1: number of protein coding genes, 2: number of rRNA genes, 3: average CAI of protein complexes) of exemplary protein complexes of the EcoCyc database. Coloring indicates significant correlations in the predicted direction (green) and in the opposite direction (red). In the third column, the number of organisms is depicted in which a homolog was found.

ComplexID (EcoCyc)	Name	#Organisms	Hypothesis 1	Hypothesis 2	Hypothesis 3

UVRABC-CPLX	UvrABC Nucleotide Excision Repair Complex	468	−0.3055	0.3972	0.1954
CPLX0-3953	30S ribosomal subunit	461	−0.0799	−0.0896	−0.0732
CPLX0-3962	50S ribosomal subunit	459	−0.0489	0.1596	0.1278
SEC-SECRETION-CPLX	Sec Holo-Translocon	456	0.0448	−0.0491	−0.1450
RNAP54-CPLX RNA	polymerase sigma 54	452	−0.1057	0.0998	−0.0241
RUVABC-CPLX	resolvasome	437	0.2518	−0.0940	−0.0101
ATPSYN-CPLX	ATP synthase/thiamin triphosphate synthase	429	−0.1624	0.2707	−0.0387
HSP70-CPLX	DnaK-DnaJ-GrpE chaperone system	397	0.0936	0.0776	−0.1871
CPLX0-1341	SufBC2D Fe-S cluster scaffold complex	347	0.0308	0.1678	−0.0322
GCVMULTI-CPLX	glycine cleavage system	344	0.2748	−0.0067	−0.2053
ACETYL-COA-CARBOXYLMULTI-CPLX	acetyl-CoA carboxylase	334	−0.0831	0.4843	−0.0148
PYRUVATEDEH-CPLX	pyruvate dehydrogenase	285	−0.2518	0.1245	−0.0722

**Table 3:** Parameter and variable overview.

Parameter	Value	Description
*u_i_*	[0,*d_i_*]	synthesis rates (decision variable)
*d_total_*	1	total synthesis capacity
*d_i_*	[0,1]	individual synthesis capacity
*k_l_*	1 or [0,2]	kinetic parameter
*T_max_*	15	timespan of production

## Supplementary Files

Supplementary File 1
