# Supplementary material for: Footprints of Optimal Protein Assembly Strategies in the Operonic Structure of Prokaryotes
Source: Metabolites. 2015 Apr 28;5(2):252–69. doi: 10.3390/metabo5020252 (PMC4495372; doi:10.3390/metabo5020252)
Supplement: Supplementary File 1 [file metabolites-05-00252-s001.zip › metabolites-05-00252-supplemetary-final/metabolites-05-00252-supplementary.pdf]

Supplementary File

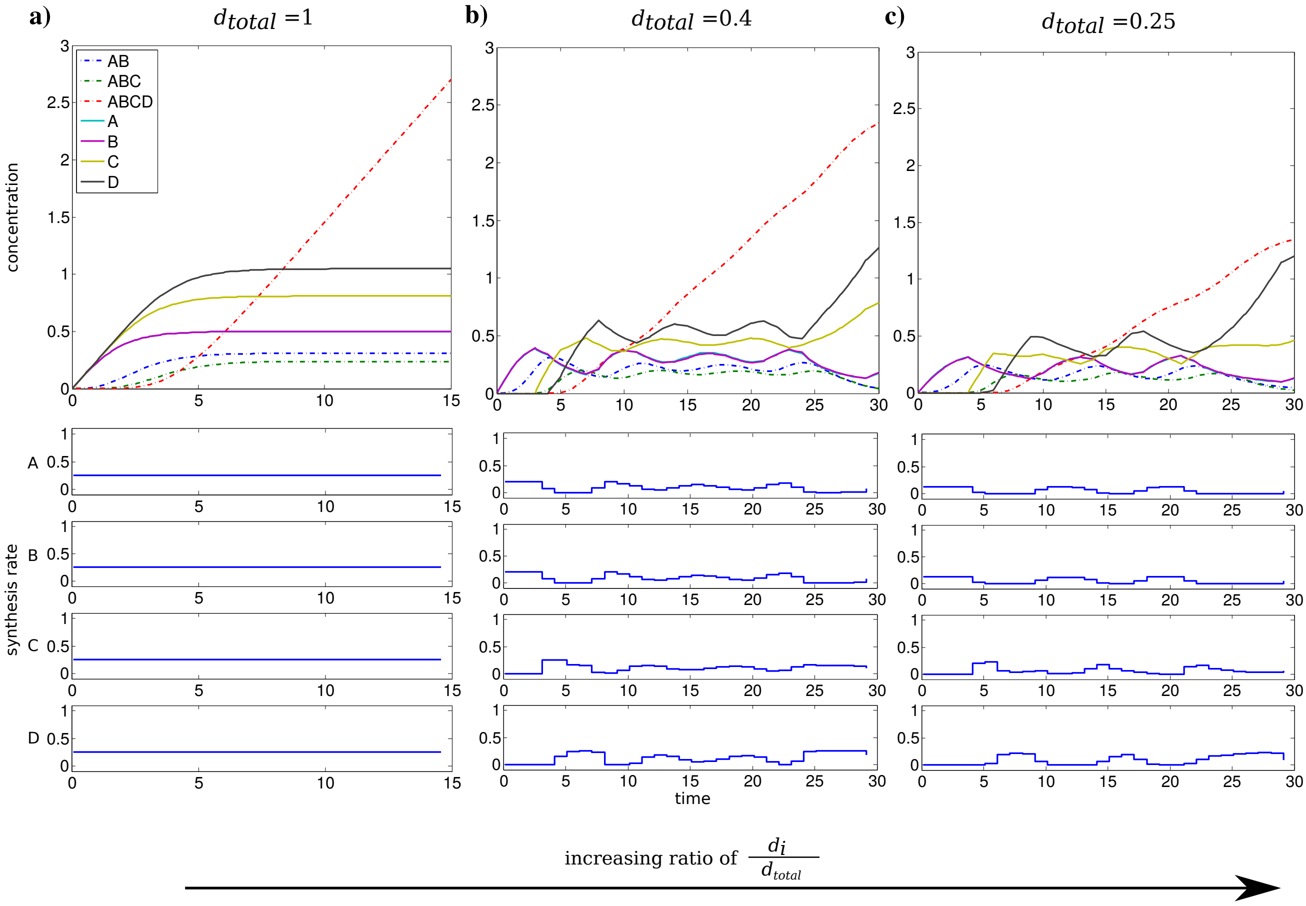

Figure S1. The figure shows protein concentrations and optimal synthesis rates for varying total synthesis capacities (**a**) 1; (**b**) 0.4 and (**c**) 0.25 while indidividual synthesis rate $d_i = 0.25$ is fixed. Results are comparable to Figure 2 and therefore we conlude, that optimal synthesis strategies are only effected by the relation of total and individual synthesis capacites.

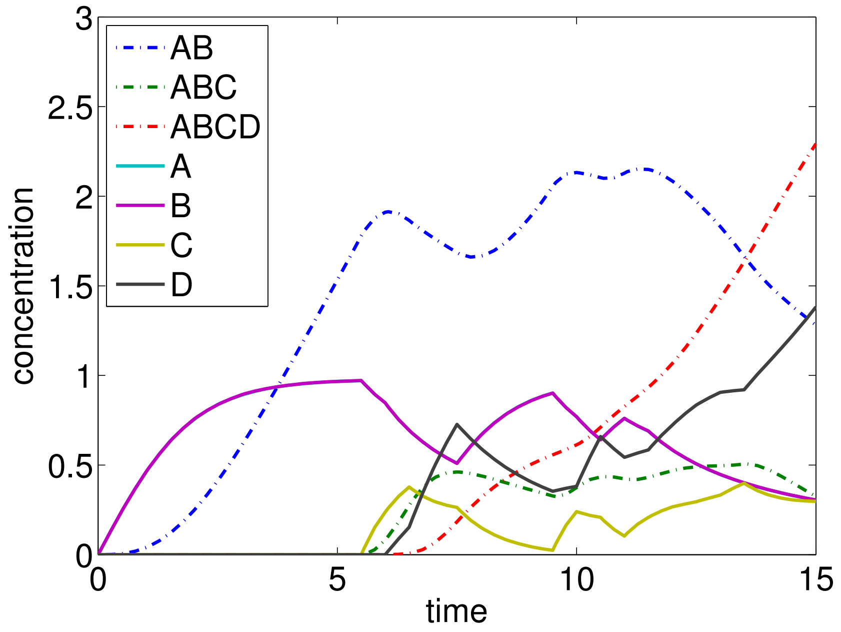

(**a**)

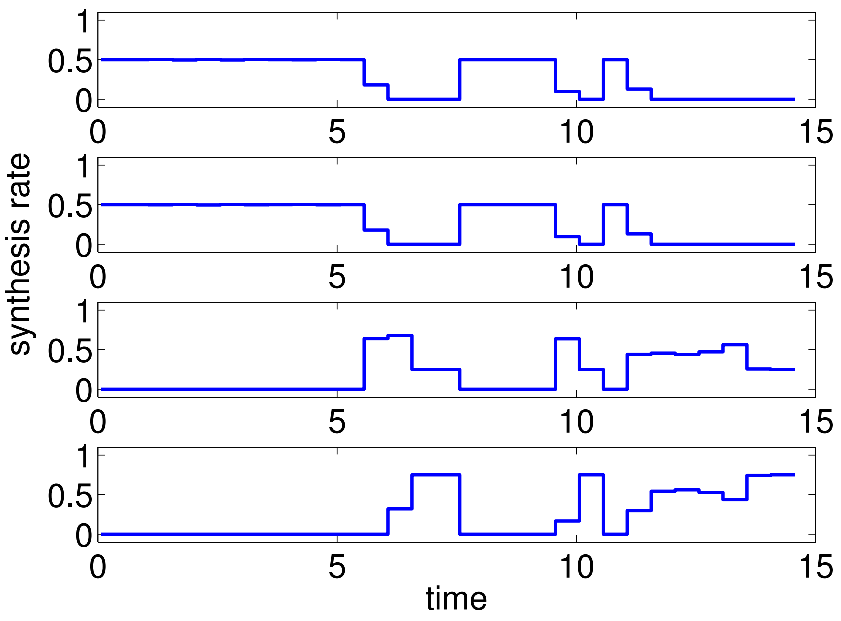

(**b**)

Figure S2. Example of longer synthesis of subunits A and B due to its slow assembly (very low k_1). (**a**) Time course of protein concentrations for the kinetic parameters: $k_1 = 0.01$, $k_2 = 1.7$, $k_3 = 1.8$; (**b**) Synthesis rates of the same parameter set.

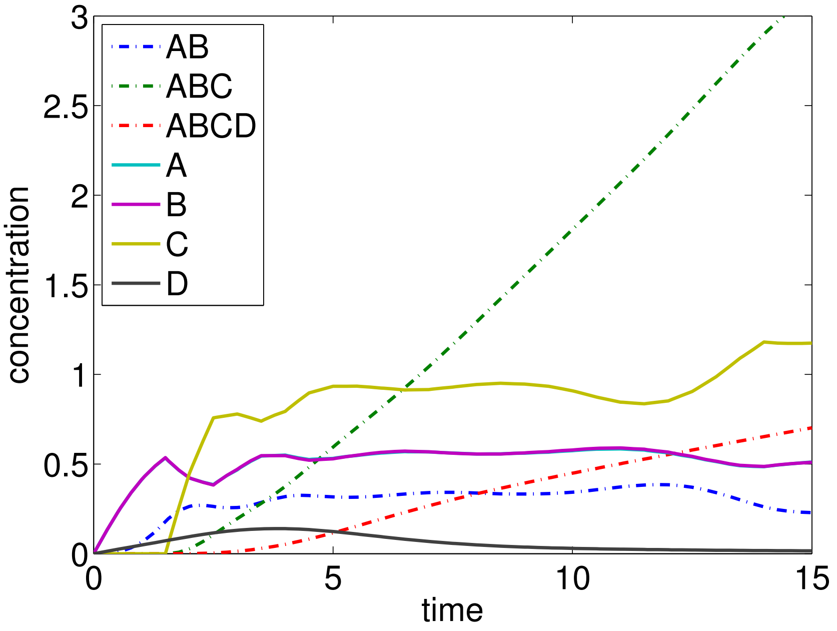

(**a**)

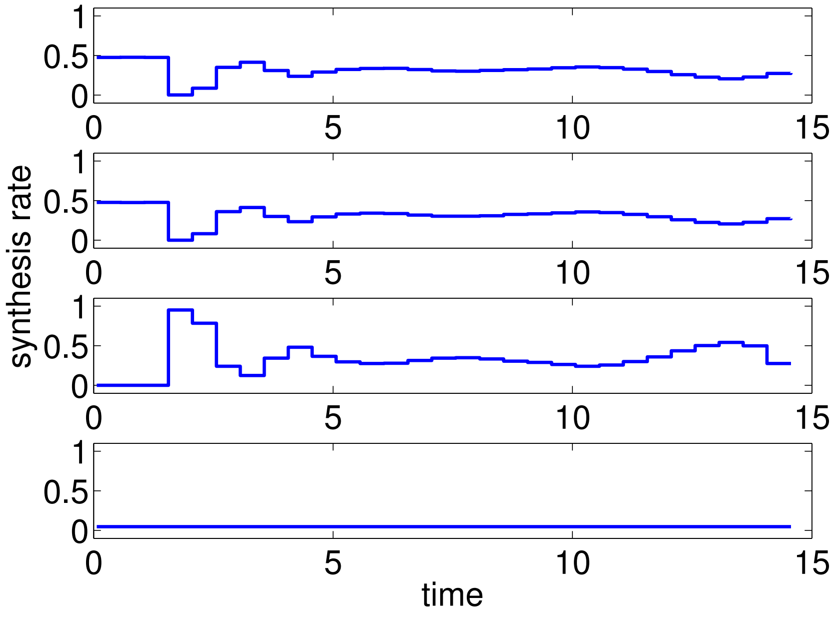

(**b**)

Figure S3. Example of early activation of subunit D due to its very low synthesis capacity d_4. (**a**) Time course of protein concentrations for synthesis capacities: $d_1 = 0.99$, $d_2 = 0.98$, $d_3 = 0.98$ and $d_4 = 0.05$; (**b**) Synthesis rates of the same parameter set.

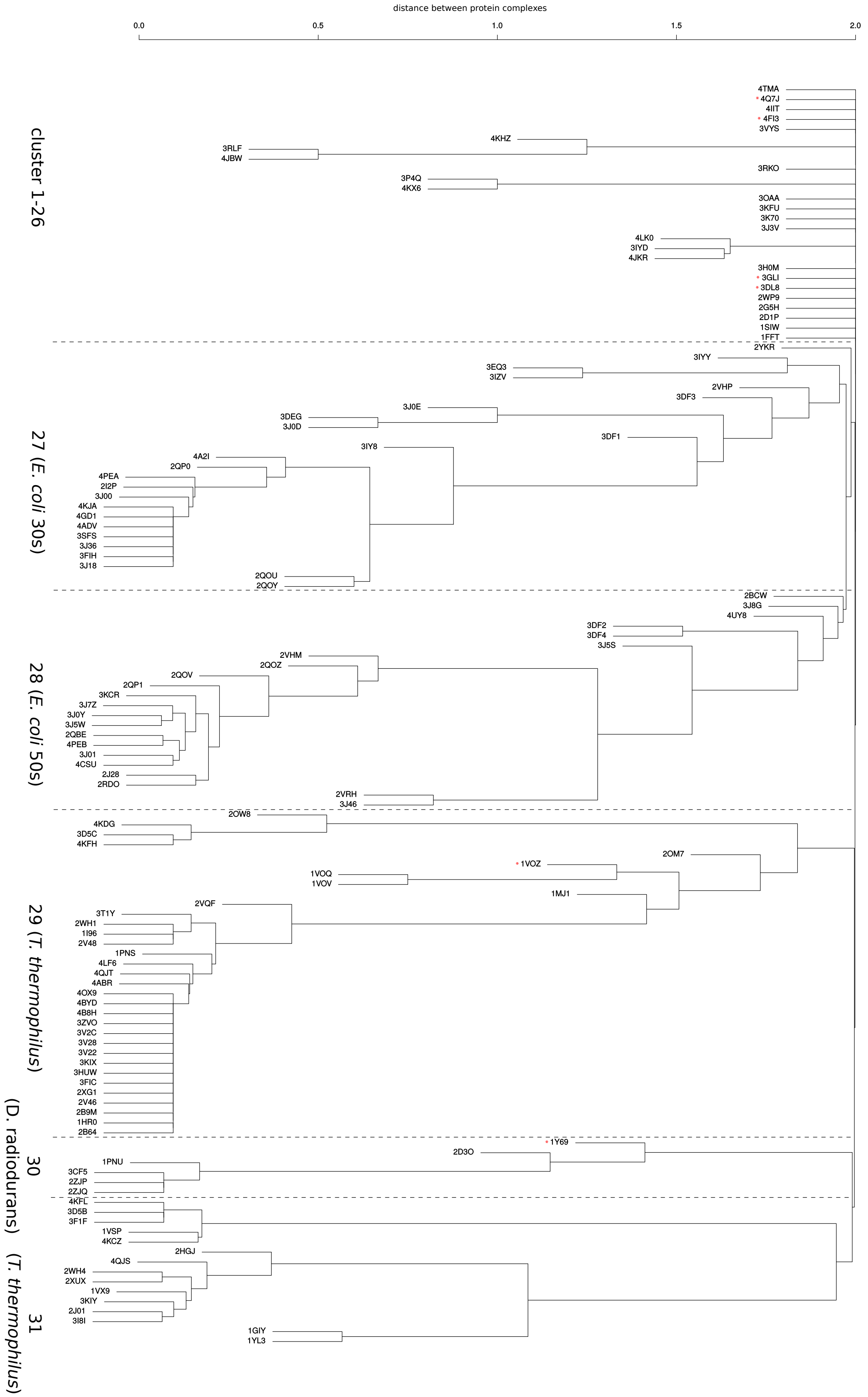

Figure S4. Dendrogramm of protein complex structures from PDB with distances calculated based on their shared subunits. The cluster assignment is given in the left and marked partially with dashed lines. For ribosomal structures the predominant organism and form is given. With * marked PDB structures are excluded in the correlation table, because they are not varying in their operon structure

Table S1. Validation results of protein complexes retrieved from the EcoCyc database. First column: protein complex ID, Second: name and general function, Third: number of organisms in which a homolog was found, following columns: partial Spearman correlation and adjusted P-value for each hypothesis.

Excel-sheet: ***eco-tableS1.ods***

Table S2. Validation results of protein complexes retrieved from the PDB database. First column: list of protein complex ID(s) combined, Second: name and general function, Third: number of organisms in which a homolog was found, following columns: partial Spearman correlation and adjusted P-value for each hypothesis.

Excel-sheet: ***pdb-tableS2.ods***
