# Supplementary figures and images for: Footprints of Optimal Protein Assembly Strategies in the Operonic Structure of Prokaryotes
Source: Metabolites. 2015 Apr 28;5(2):252–69. doi: 10.3390/metabo5020252 (PMC4495372; doi:10.3390/metabo5020252)

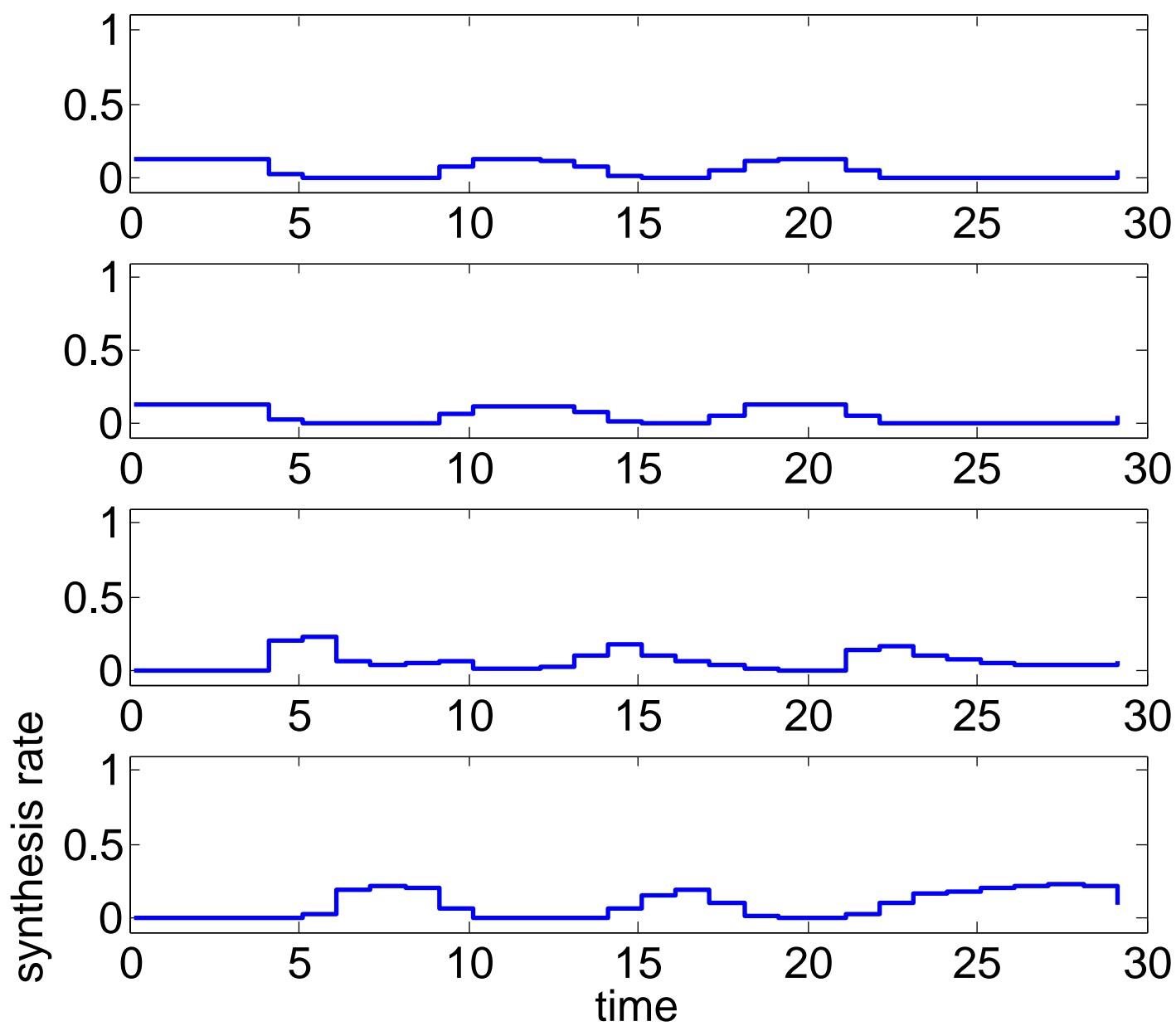

Supplement: Supplementary File 1 [file metabolites-05-00252-s001.zip › metabolites-05-00252-supplemetary-final/SupplementData/activation_dtot025.pdf]

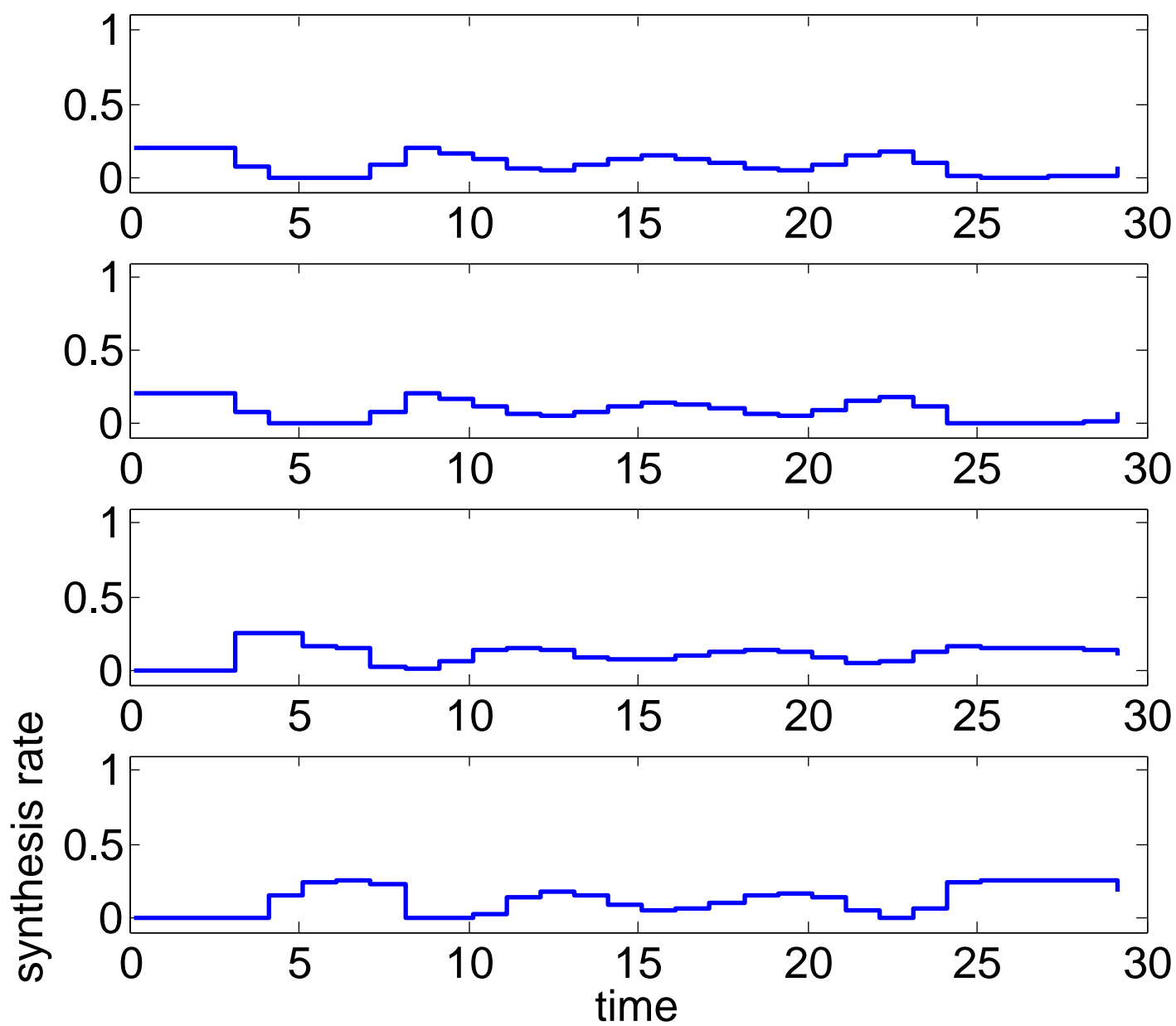

Supplement: Supplementary File 1 [file metabolites-05-00252-s001.zip › metabolites-05-00252-supplemetary-final/SupplementData/activation_dtot04.pdf]

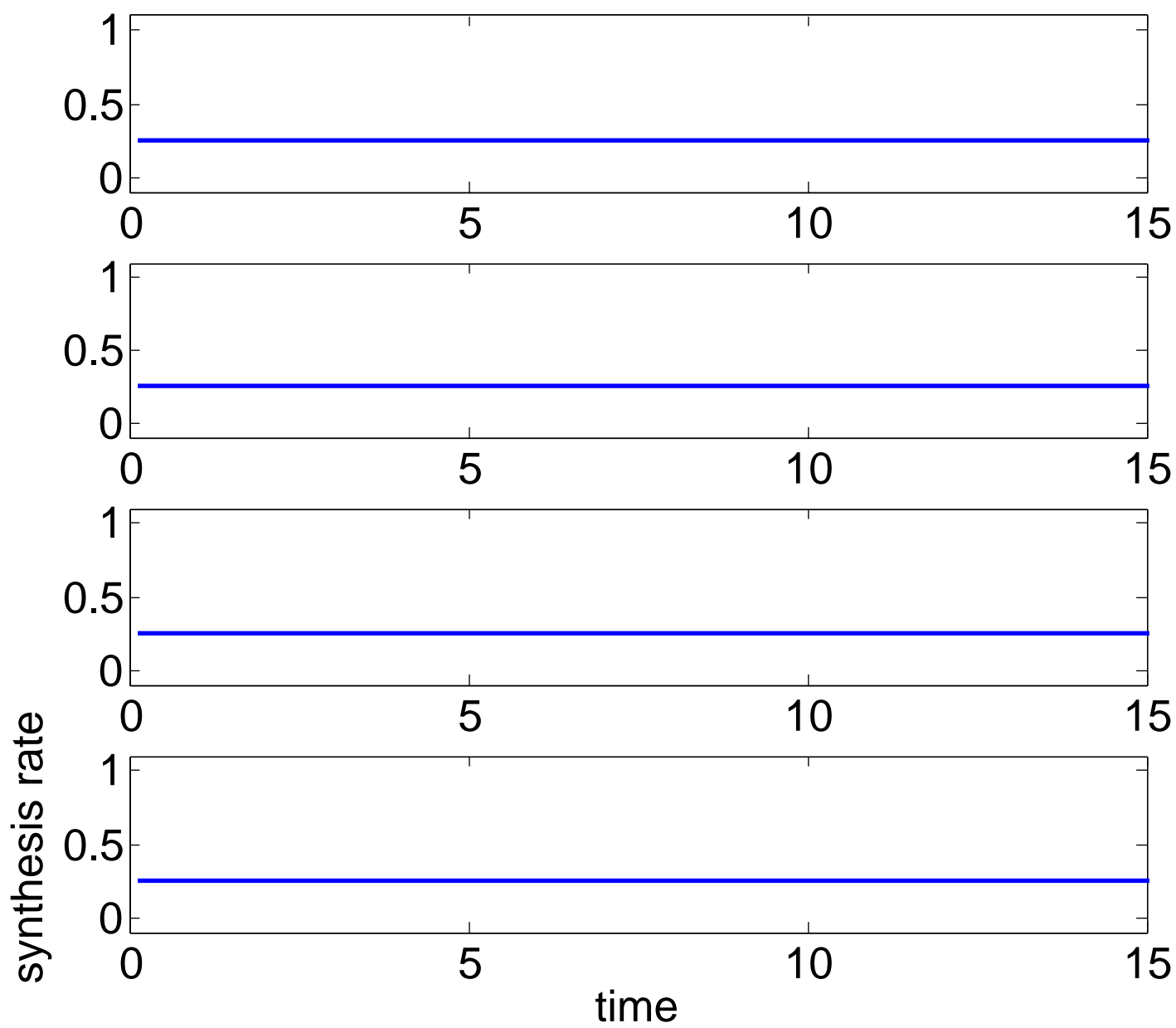

Supplement: Supplementary File 1 [file metabolites-05-00252-s001.zip › metabolites-05-00252-supplemetary-final/SupplementData/activation_dtot1.pdf]

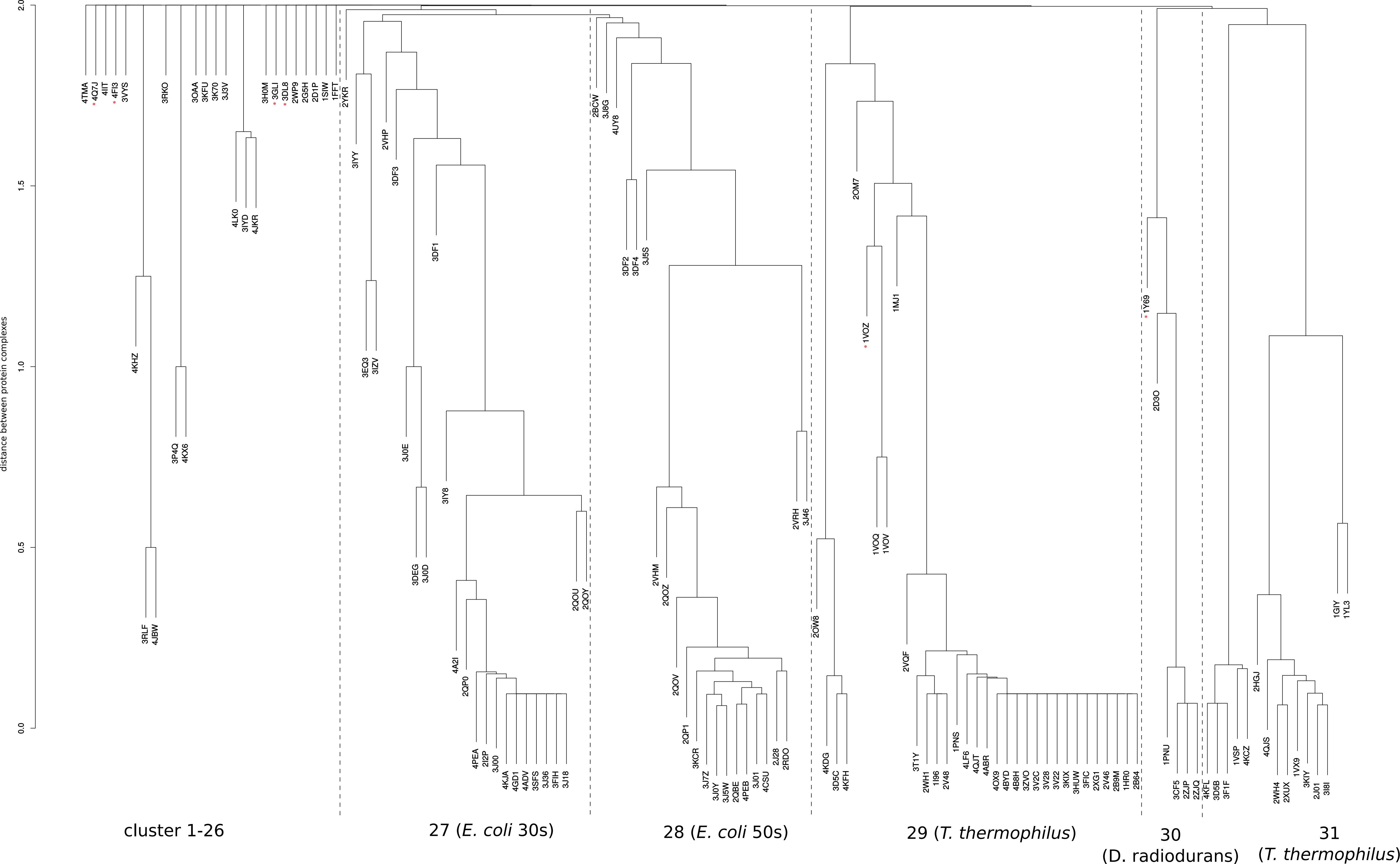

Supplement: Supplementary File 1 [file metabolites-05-00252-s001.zip › metabolites-05-00252-supplemetary-final/SupplementData/dendrogramm.pdf]

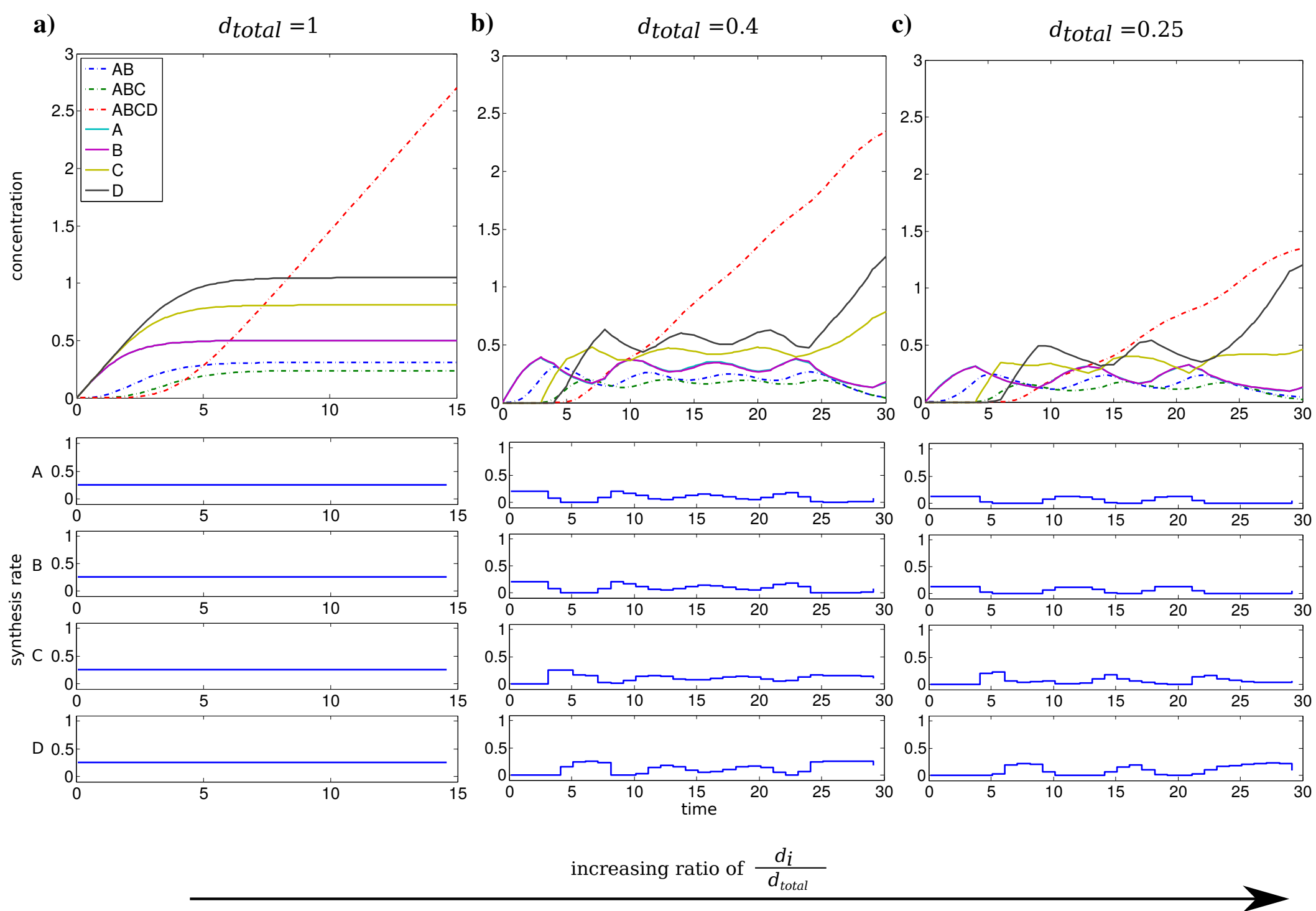

Supplement: Supplementary File 1 [file metabolites-05-00252-s001.zip › metabolites-05-00252-supplemetary-final/SupplementData/ind_vs_total_ratio_dtotal.pdf]

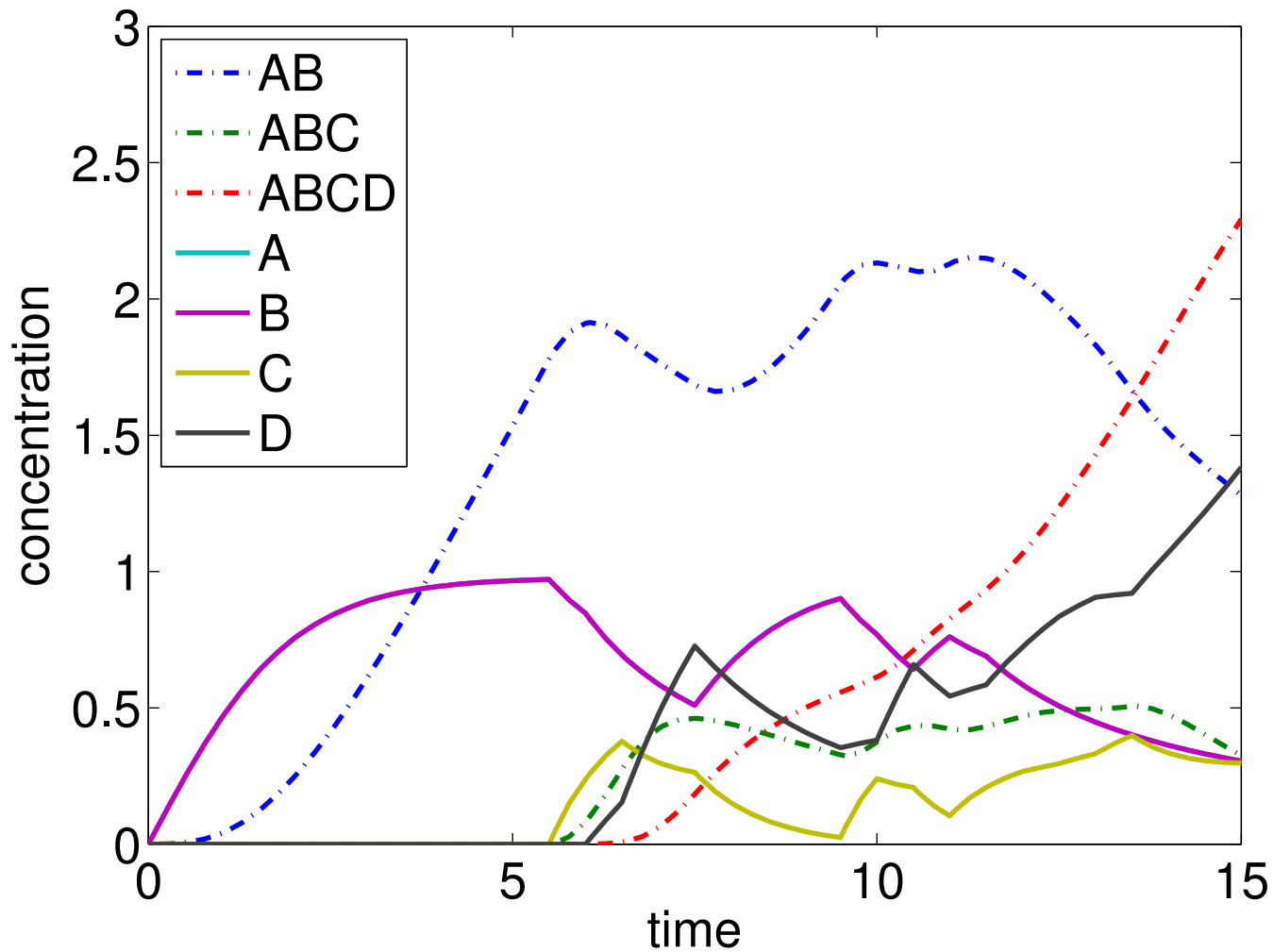

Supplement: Supplementary File 1 [file metabolites-05-00252-s001.zip › metabolites-05-00252-supplemetary-final/SupplementData/kin_example_conc.pdf]

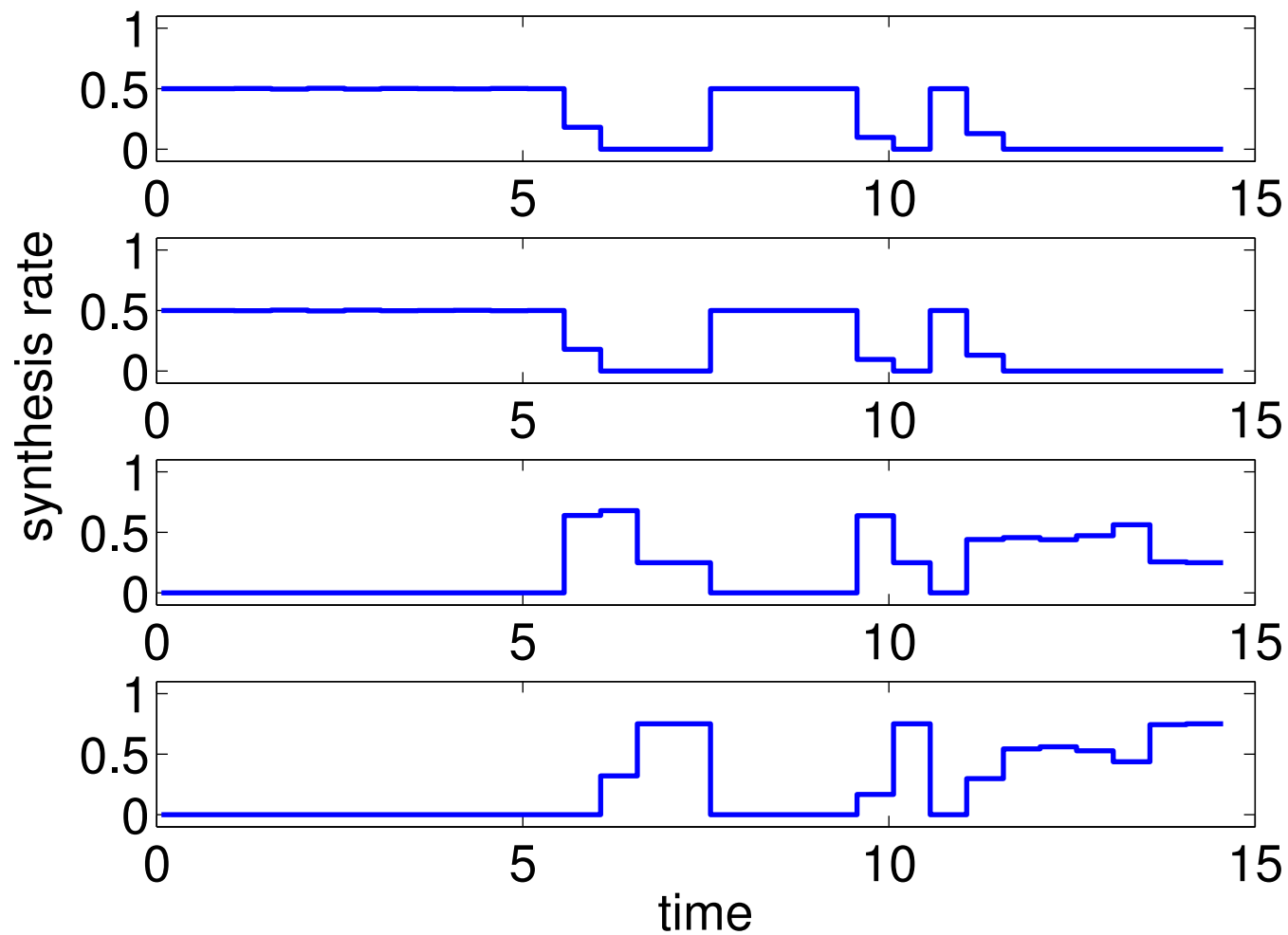

Supplement: Supplementary File 1 [file metabolites-05-00252-s001.zip › metabolites-05-00252-supplemetary-final/SupplementData/kin_example_rate.pdf]

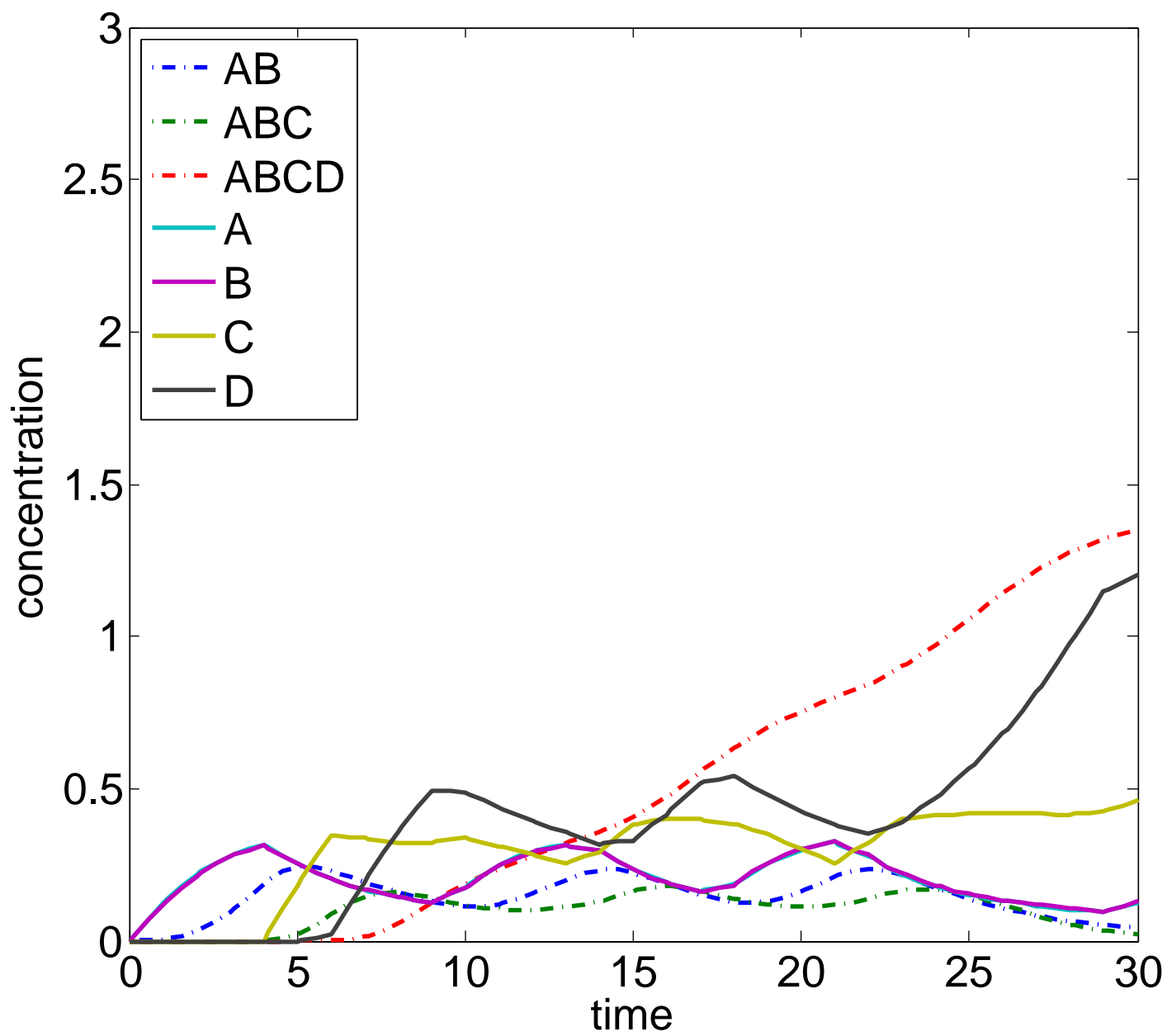

Supplement: Supplementary File 1 [file metabolites-05-00252-s001.zip › metabolites-05-00252-supplemetary-final/SupplementData/time_dtot025.pdf]

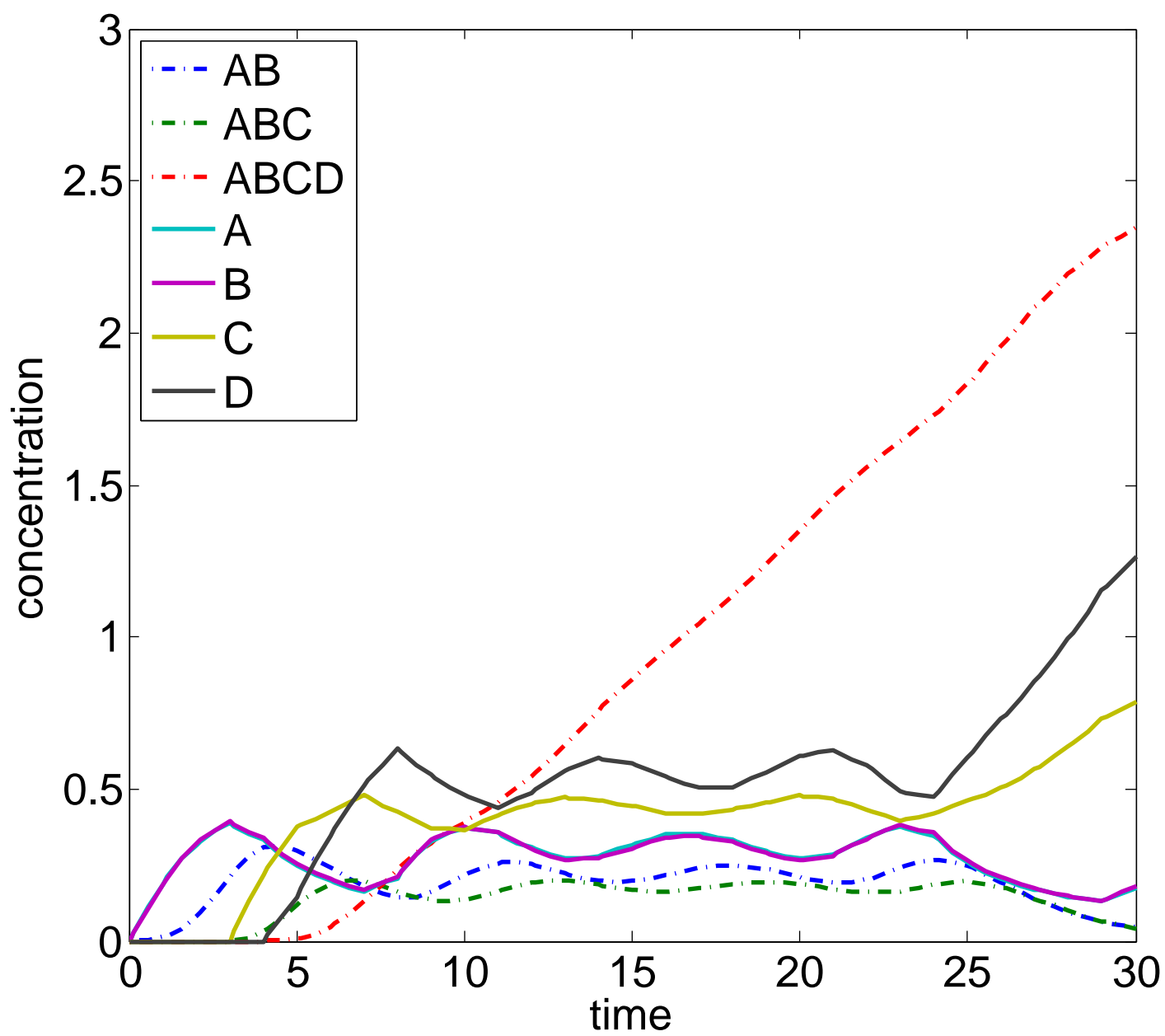

Supplement: Supplementary File 1 [file metabolites-05-00252-s001.zip › metabolites-05-00252-supplemetary-final/SupplementData/time_dtot04.pdf]

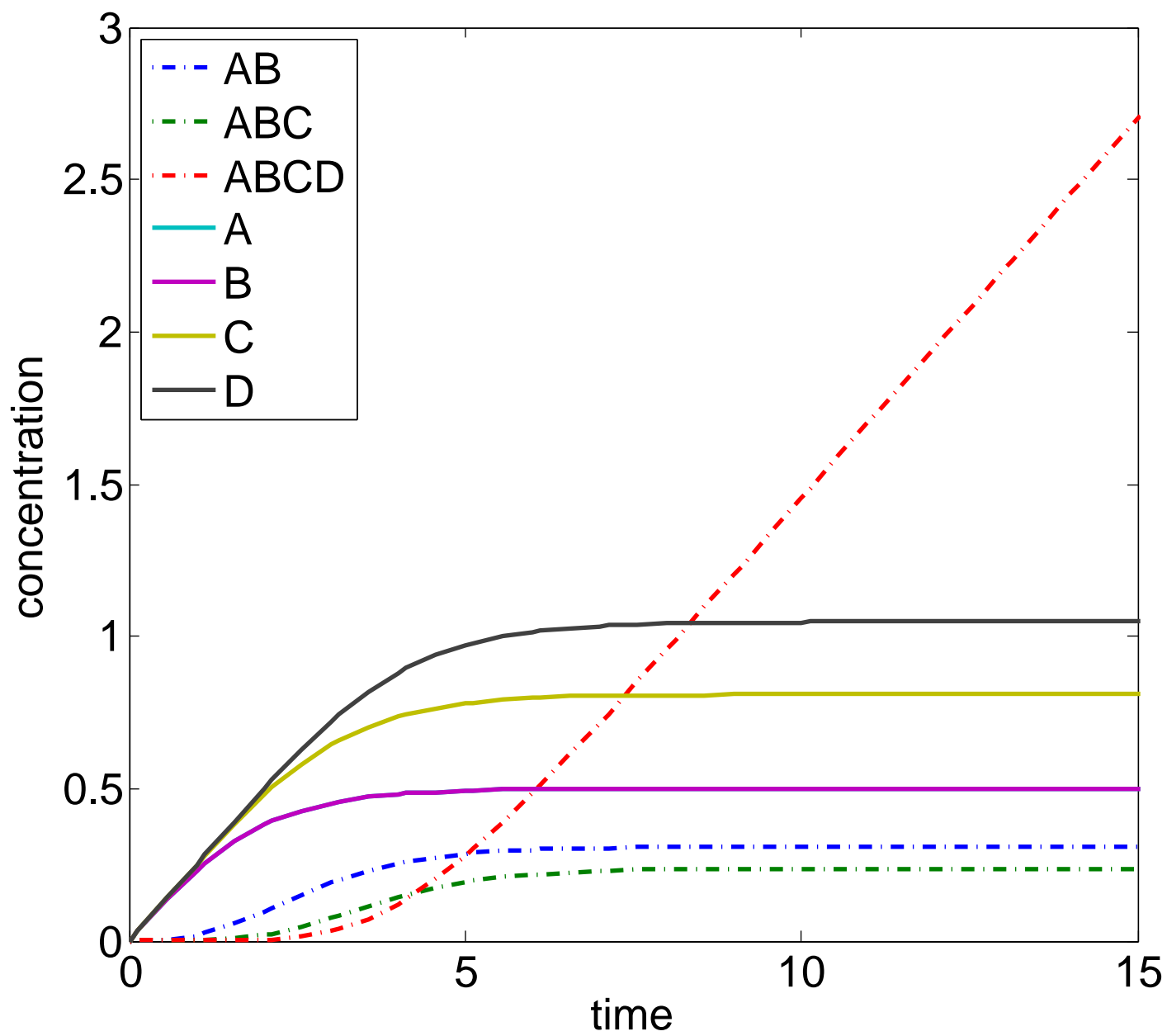

Supplement: Supplementary File 1 [file metabolites-05-00252-s001.zip › metabolites-05-00252-supplemetary-final/SupplementData/time_dtot1.pdf]

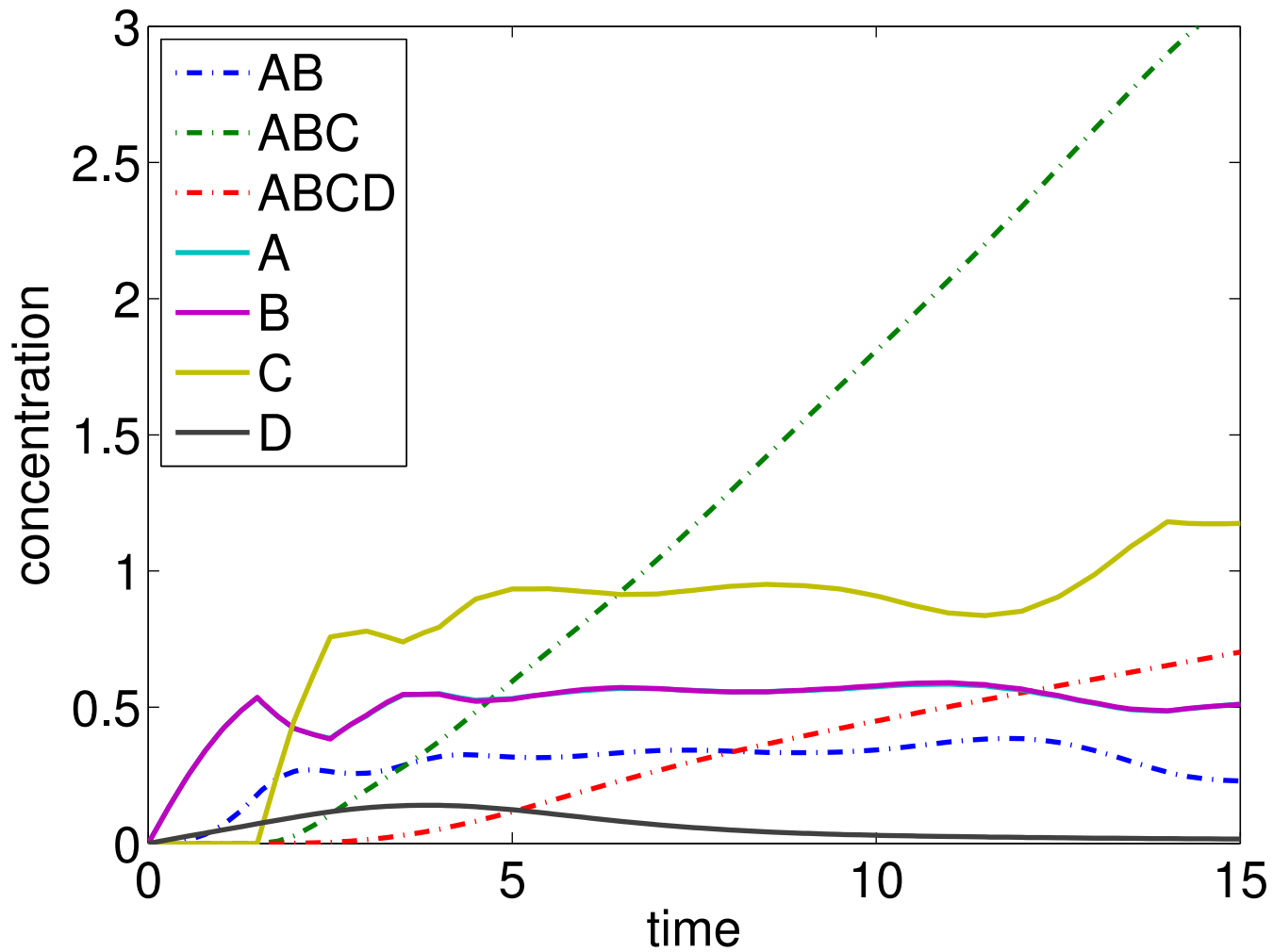

Supplement: Supplementary File 1 [file metabolites-05-00252-s001.zip › metabolites-05-00252-supplemetary-final/SupplementData/varDi_example_conc.pdf]

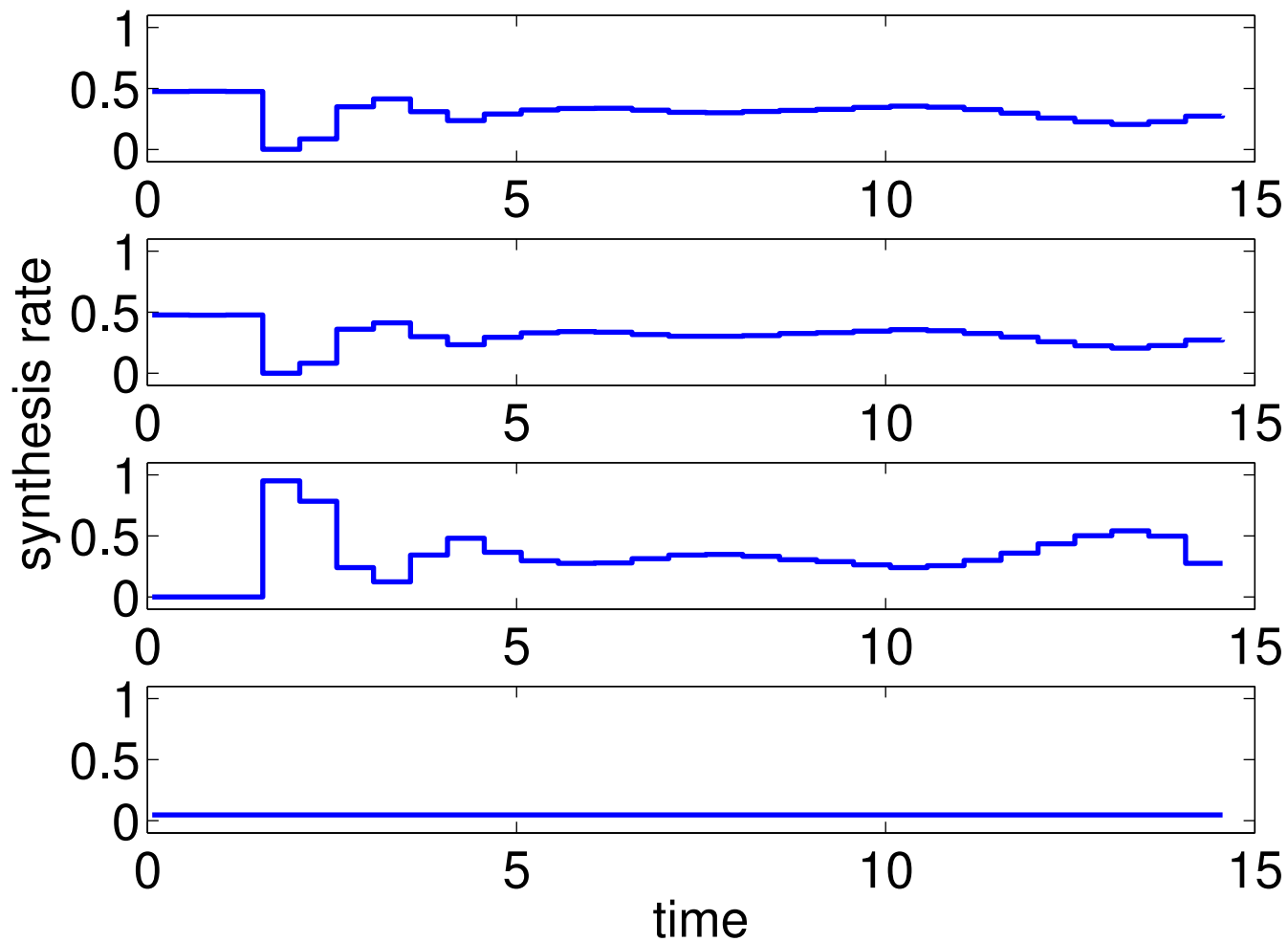

Supplement: Supplementary File 1 [file metabolites-05-00252-s001.zip › metabolites-05-00252-supplemetary-final/SupplementData/varDi_example_rate.pdf]
